# Piecewise Parameter Optimization of the Neo-Hookean Model for Hyperelastic Silicone Rubber with Large Deformation

**DOI:** 10.3390/ma19132789

**Published:** 2026-07-01

**Authors:** Ruibing Fan, Pengyu Xu, Yao Wang, Guowei Shao, Jianhua Tang

**Affiliations:** 1 School of Intelligent Manufacturing, Changzhou Vocational Institute of Textile and Garment, Changzhou 213164, China; rbfan@cztgi.edu.cn (R.F.); jdywang@cztgi.edu.cn (Y.W.); gwshao@cztgi.edu.cn (G.S.); jhtang@cztgi.edu.cn (J.T.); 2 Jiangsu Research Center of Intelligent Manufacturing Technology for Carbon Fiber and Advanced Material, Changzhou 213164, China; 3 Engineering Research Center of Artificial Intelligence for Textile Industry Ministry of Education, Institute of Artificial Intelligence, Donghua University, Shanghai 201620, China; 4 School of Material Science and Engineering, Zhengzhou University, Zhengzhou 450001, China

**Keywords:** silicone rubber, nonlinear, soft actuator, piecewise, Neo-Hookean model

## Abstract

Soft actuators are increasingly being used in robotics and biomedical applications. They use hyperelastic materials, such as silicone rubber, to generate large reversible deformations. However, it is not easy to model the mechanical behavior of silicone rubber under large deformations. It is difficult to accurately predict its nonlinear hyperelastic behavior and thus to accurately design and control these actuators. We have created an optimized Neo-Hookean constitutive model of Ecoflex 00-30 silicone rubber. This method is based on the combination of theory and experiments, whose goal is to enhance the usefulness of the model for performance analysis of soft actuators. Dumbbell-shaped specimens were tested in uniaxial tension on a ZQ-990LB testing machine in a controlled environment at 25.4 °C and 57.4% relative humidity (RH). Stretch ratios varied between 1 and 8.6 and tensile speeds up to 500 mm/min were used. Stress–strain curves and fracture behavior were captured by the experiments. The Neo-Hookean model was then fitted and optimized using a global least-squares optimization approach. Two changes were made: piecewise segmentation of the data, and variable weight factors for uniaxial and equibiaxial tensile data. This accuracy was better for each of the stretch ratios. The optimized material parameters yielded curves that were in close agreement to the experimental data—significantly better than fitting using conventional single regime, particularly in each of the segmented ranges. The model breaks the range of deformations into segments, and in each segment it reflects the response of the silicone rubber to the various loadings. The results provide a good theoretical foundation for modeling the mechanics, analyzing the kinematics and developing intelligent control strategies for pneumatic soft actuators. This should help propel their engineering applications in dynamic environments.

## 1. Introduction

Per structure, actuators can be of two types, rigid and soft. The rigid actuators typically work on linkage mechanisms, where consecutive links are joined by kinematic pairs. The latter are rigid during movement and do not exhibit any significant movement-related deformations [1]. Soft actuators go the other way. They are composed of flexible materials or flexible structures; thus, their motion relies on the material properties and structures. Such coupling frequently generates very large deformations in the mechanism of actuation and the whole structure when the mechanism is working. Based on different flexible materials and actuation structures, soft actuators have a variety of actuation modes, including shape memory alloy actuation, pneumatic or hydraulic actuation, artificial muscle actuation, electro-active polymer actuation and biological gel actuation.

Hyperelastic materials such as silicone rubber have the ability to undergo large strains and recover well, and for this reason, silicone rubber widely used in pneumatic soft actuators [2]. Silicone rubber soft actuators are continuum deformation actuators, that is, there are no discrete linkage mechanisms and no kinematic pairs, so they have high degrees of freedom. Any portion of the actuator can be bent, making it suitable for complex and unstructured environments. Due to the simplicity, high speed, low cost, and reliable actuation energy of pneumatic actuation methods, researchers have extensively studied and applied pneumatic soft actuators based on pneumatic artificial muscles. OK Afsar reported electrofluidic fiber muscles—soft, electrically driven, untethered artificial muscles—that achieve skeletal-muscle-like power density, strain, and speed through antagonistic fluidic actuators and electrohydrodynamic fiber pumps [3]. The soft brake used OmniFiber, a soft, line-based fluidic material system comprising thin (<1.8 mm) actuated fibers with integrated soft sensors that deliver forces up to 19 N and linear actuation speeds of 150 mm/s, enabling woven, interactive morphing applications such as on-body haptic devices and synchronized tangible interfaces [4].

Unlike the conventional rigid materials, most silicone rubbers do not possess well-defined mechanical models or documented parameters of their material properties. The manufacturers usually provide basic characteristics such as Shore hardness and tensile strength, but not experimental characterization of the mechanical behavior of the material. So, raw data for determining mechanical model parameters are hard to come by [5,6].

How soft actuators deform and move is directly controlled by the hyperelasticity and nonlinear mechanical properties of silicone rubber. To ensure their deformation accuracy and control precision, it is important to study their mechanical behaviors both theoretically and experimentally [7,8].

Silicone rubber is a polymer which is highly transparent, heat resistant and safe (non-toxic). The Poisson’s ratio is in the range 0.4–0.499 and the material is isotropic. It has been observed to have large, nonlinear deformation. It has an elastic modulus of 1–6 MPa and its elongation at break is more than 900%. For theoretical modeling and engineering use of soft actuators, the mechanical properties of silicone rubber need to be tackled first. Hyperelasticity, viscoelasticity and the Mullins effect are the main characteristics to consider [9].

To analyze the mechanical behavior of silicone rubber, a suitable constitutive model has to be set up. There are two types of these models. The first one is the statistical thermodynamic model that describes the macroscopic properties, starting from the molecular chain network structure. The second one is the phenomenological model which is based on the strain energy function. This method involves fitting material parameters to experimental data gained from mechanical tests to describe the behavior of silicone rubber [10,11,12].

Mooney developed a scalar function that describes the internal energy changes in silicone rubber, but his strain energy function omits the volume change and strain hysteresis of the rubber under deformation [13]. To account for changes in shear modulus in the deformation, Yeoh added an exponentially decaying term [14]. Marckmann and Verron tested 20 different constitutive models and fitted tensile test data using them [15]. Beomkeun selected several strain energy functions and fitted the experimental data, obtaining a constitutive model which reflects the mechanical properties of Neoprene rubber [16]. Steinmann et al. developed several constitutive models and determined strain energy functions that work for uniform internal deformation [17]. Popular constitutive models for silicone rubber are the Mooney–Rivlin, Yeoh, Ogden, Gent, and Neo-Hookean models. Stress–strain relations are captured by these models as a strain energy function, which provides a foundation for mechanical modeling, simulation and engineering efforts.

Silicone rubber exhibits complex behavior of all mechanical properties, including hyperelasticity, viscoelasticity and the Mullins effect, as a hyperelastic material. Thus, its stress–strain behavior is nonlinear and the material is different for various ranges of strain. Various strategies have been developed to fit hyperelastic constitutive models to experimental data spanning a wide range of stretches, motivated by the fact that a single parameter set fitted globally often fails to capture the distinct stiffening, plateau, and lock-up regions simultaneously. Ogden’s multi-term strain energy formulation offered an early and implicit solution to this problem: different series terms were tailored to govern the response in different portions of the stretch domain, and the parameter estimation was later refined through nonlinear optimization with attention to non-uniqueness [18]. A more radical departure was taken by Sussman and Bathe, who abandoned parametric fitting altogether and instead constructed the strain energy through piecewise cubic spline interpolation of experimental data [19]. Latorre and Montáns extended this philosophy to anisotropic materials under the WYPIWYG framework [20,21]. Anssari-Benam et al. introduced the generalized Mooney space as a diagnostic tool that transforms the fitting problem into a well-conditioned coordinate system, thereby revealing regime-dependent trends obscured in conventional stress–stretch representations [22]. On a broader scale, Ricker and Wriggers conducted a systematic evaluation of nine rubber compounds, providing practical guidance on data preprocessing, cost function selection, and invariant-dependent calibration [23]. Dal et al. demonstrated a data-driven strategy that embeds B-spline representations directly into the strain energy function while enforcing constitutive stability constraints [24]. In real applications, soft actuator structures frequently use different silicone rubbers with varying elastic moduli such that each silicone rubber operates within a different stress/strain range. Hence, it is necessary to investigate the mechanical properties through both theoretical modeling and experimental analysis. Building on these works, the present study proposes a pragmatic engineering approach that extends the usable range of the simple Neo-Hookean model through piecewise parameter optimization with tunable weight factors for uniaxial and equibiaxial tension contributions. Unlike the staged model-selection approach of Destrade et al., we retain a single model form (Neo-Hookean) across all deformation regimes while allowing the effective shear modulus to vary with stretch, which maintains compatibility with standard FEM implementations. Unlike the comprehensive multi-model studies of Marckmann and Verron and Steinmann et al., our focus is on providing a practical, implementation-ready parameter set for soft actuator designers who require a simple constitutive description that works across the full deformation range of Ecoflex 00-30. The piecewise weighted optimization strategy thus offers an intermediate approach between single-regime simple models and more complex unified models, balancing accuracy, simplicity, and ease of implementation.

The mechanical properties of silicone rubber, the basic characteristics of the material, hyperelastic constitutive models, and the uniaxial tensile testing experiments we conducted are discussed here. While the Neo-Hookean model is one of the simplest hyperelastic formulations, its standard single-regime fitting often yields poor accuracy across the large deformation ranges encountered in soft actuator applications. In this study, we propose a practical engineering approach that extends the usable range of the Neo-Hookean model through piecewise parameter optimization with tunable weight factors for uniaxial and equibiaxial tension contributions. The key practical advantage of this approach is that the resulting piecewise parameters can be directly implemented in any finite element code that supports the Neo-Hookean model—which includes virtually all commercial and open-source FEM packages—without requiring user-defined material subroutines. This bridges the gap between accurate hyperelastic characterization and practical actuator design, offering a readily usable tool for soft robotics practitioners who need a simple constitutive description that works across the full deformation range of Ecoflex 00-30 silicone rubber. The present study focuses on quasi-static, hyperelastic behavior; viscoelasticity, the Mullins effect, fracture, and rate-dependent behavior are not incorporated in the current model.

## 2. Constitutive Model of Silicone Rubber

### 2.1. Material Properties of Silicone Rubber

Hyperelastic materials show up in soft actuators as both structural components and actuation elements. Their intrinsic properties shape how these devices deform and move. Actuator stiffness is usually described by Young’s modulus. For rigid actuators, the structural materials—metals, plastics, or synthetic composites—have a Young’s modulus above 1 GPa. For soft actuators, the modulus sits below 1 GPa. Rubber, silicone rubber, and gels are the most common choices here [25,26]. Soft actuators built from flexible materials have a high degree of freedom, deform easily, and adapt well to changing environments.

A flexible material’s properties essentially set the actuation characteristics of the soft actuator it goes into. Take silicone rubber as an example. It gets used a lot as the actuation material for pneumatic soft actuators because it is elastic, recovers well, is safe, and stays chemically stable. Hyperelastic silicone rubbers include the Ecoflex series, the Dragon Skin series, and Sylgard 184. In this study, Ecoflex 00-30, a hyperelastic silicone rubber, was selected as the actuation material for the pneumatic soft actuator. Ecoflex 00-30 is a platinum-cured silicone rubber that exhibits excellent flexibility, with an elongation at break of up to 1000%, and is classified as an RTV-2 silicone rubber. Compared with conventional sulfur-cured silicone rubbers, Ecoflex silicone rubber undergoes hydrosilylation during the curing process, in which hydrogen-containing silicone oils react with vinyl double bonds. This reaction prevents the formation of new low-molecular-weight structures. As a result, the material becomes hygienic and environmentally friendly. This silicone rubber has a low curing temperature. It also offers fast curing speed. It possesses excellent physical and chemical stability. Moreover, it exhibits superior elasticity and recoverability. Ecoflex series silicone rubbers remain liquid at room temperature; their molding and curing require mixing components A and B in a 1:1 volume ratio, followed by curing at room temperature or elevated temperatures. Once cured, the silicone rubber exhibits high elongation at break (up to 1000%), full recovery after unloading, and elastic behavior over a temperature range of –60 to 300 °C. Table 1 presents the physical parameters of the silicone rubbers. The test data reported in the table were supplied by Smooth On.

As a hyperelastic material, silicone rubber exhibits substantial elasticity and recoverability, which makes it challenging to establish a precise mechanical model. For the Ecoflex series of silicone rubbers, a combination of experimental analysis and theoretical modeling is required to investigate their mechanical properties. We built a constitutive model for this study to capture the mechanical behavior of silicone rubber, focusing on its hyperelastic response. When you apply stress to silicone rubber, the material deforms elastically by a large amount. The mechanical work done on it turns entirely into strain energy and stays stored inside. Take the stress off, and it returns to its original shape.

At the molecular level, the polymer chains in silicone rubber are crosslinked uniformly, so the material behaves isotropically. This means we can describe its mechanical behavior with an elastic strain energy function per unit volume. The constitutive model gives us the foundation for analyzing the mechanical properties of silicone rubber—it establishes the link between the deformation gradient and the strain energy function when stress acts on the material. The stress–strain relationship of silicone rubber is nonlinear; in this study, this relationship was derived from the strain energy function.

### 2.2. Hyperelastic Constitutive Model

A wide range of hyperelastic constitutive models has been developed to describe the mechanical behavior of rubber-like materials, including the Mooney–Rivlin, Yeoh, Ogden, Gent, and Neo-Hookean models [11,12,13,14,15]. Each of these models offers different trade-offs between mathematical complexity, accuracy across deformation ranges, and ease of numerical implementation [27]. For the present study, the Neo-Hookean model was selected as the constitutive model for Ecoflex 00-30 silicone rubber. This choice is motivated by two considerations: (i) the Neo-Hookean model provides a reasonable first-order approximation of hyperelastic behavior with a single material parameter (the shear modulus μ), making it straightforward to calibrate; and (ii) the Neo-Hookean model is natively implemented in virtually all commercial and open-source finite element codes, facilitating its direct use in soft actuator simulation without requiring user-defined material subroutines. The general hyperelastic formulation presented below provides the theoretical foundation from which the Neo-Hookean model is derived as a special case and establishes the assumptions that underlie the subsequent derivation. This function is written as W per unit volume:

(1) $$W=W(F)=W(C)=W\left(I_{1},I_{2},I_{3}\right)$$

Here, F denotes the deformation gradient tensor, and *tr*(*F^T^F*) is the strain tensor invariant. In the deformed state of silicone rubber, the invariants of the strain tensor differ from those of the stress tensor. For isotropic materials, W depends on C through its invariants. The three principal invariants of C are defined as

(2) $$I_{1}=tr(C)=tr(F^{T}F)$$

(3) $$I_{2}=\frac{1}{2}\left[{\left(tr(C)\right)}^{2}-tr(C^{2})\right]$$

(4) $$I_{3}=\det(C)={\left(\det(F)\right)}^{2}=J^{2}$$

Here, I_1_—first principal invariant, sum of the diagonal components of C; I_2_—second principal invariant, area measure of deformation; and I_3_—third principal invariant, volume measure of deformation [28]. They represent the first, second, and third invariants, respectively. Since the deformation of silicone rubber falls within the regime of large deformation, a precise description of the relationship between strain and displacement is required, and the higher-order terms of relative displacement cannot be neglected. Using the Lagrangian description, the strain tensor invariants can be expressed in terms of the principal stretch ratios:

(5) $$I_{1}=\lambda_{1}^{2}+\lambda_{2}^{2}+\lambda_{3}^{2}$$

(6) $$I_{2}=\lambda_{1}^{2}\lambda_{2}^{2}+\lambda_{2}^{2}\lambda_{3}^{2}+\lambda_{1}^{2}\lambda_{3}^{2}$$

(7) $$I_{3}=\lambda_{1}^{2}\lambda_{2}^{2}\lambda_{3}^{2}=J^{2}$$

Here, *λ*_1_, *λ*_2_, and *λ*_3_ represent the three principal stretch ratios of the material [29]. *J* is the determinant of the deformation gradient. For silicone rubber, the bulk modulus K is much larger than the shear modulus μ, so the material can be treated as incompressible:

(8) $$J=\det(F)=\lambda_{1}\cdot \lambda_{2}\cdot \lambda_{3}=1$$

(9) $$I_{3}=J^{2}=1$$

Under incompressibility, *W* depends only on *I*_1_ and *I*_2_, and the hydrostatic pressure p becomes an indeterminate Lagrange multiplier determined by boundary conditions. The Mooney–Rivlin model is a two-parameter phenomenological model that captures the moderate-strain behavior of rubber-like materials:

(10) $$W=C_{10}(I_{1}-3)+C_{01}(I_{2}-3)+\frac{1}{2}K{\left(J-1\right)}^{2}$$

Here, *C*_10_, *C*_01_ represent Mooney–Rivlin material constants [Pa]; For an incompressible material, *J* = 1, the volumetric term vanishes:

(11) $$W=C_{10}\left(I_{1}-3\right)+C_{01}\left(I_{2}-3\right)$$

### 2.3. Neo-Hookean Constitutive Model

Based on the material characteristics of the Ecoflex series, the Neo-Hookean model was adopted as the constitutive model for the silicone rubber in the soft actuator. In this model, the strain energy density is dependent on the strain tensor invariant *tr*(*F^T^F*). Through this formulation, a quantitative relationship is established between the specific strain energy *W* and the stretch ratios. The following expression defines the strain energy density for the Neo-Hookean model:

(12) $$W=\frac{\mu }{2}\left(I_{1}-d\right)+\frac{K}{2}{\left(J-1\right)}^{2}$$

(13) $$K=\frac{E\nu }{\left(1+\nu \right)\left(1-2\nu \right)}$$

Here, *μ* is the initial shear modulus as a constant, *J* is the volume ratio, for incompressibility logJ = 0, and *d* is a parameter related to material compressibility [30]. d is a compressible parameter with shifting resting state, d = 3 [31]. Young’s modulus E and Poisson’s ratio *ν* determine the values of the shear modulus *μ* and the bulk modulus *λ*. The Neo-Hookean strain energy function adopted in this study follows the incompressible formulation (J = 1), which rigorously corresponds to a Poisson’s ratio of ν = 0.5. This incompressible assumption is consistent with the physical behavior of silicone rubber under moderate deformations, where the volumetric change is negligible. However, when implementing incompressible or nearly incompressible hyperelastic models in standard finite element codes, a fully incompressible formulation (ν = 0.5 exactly) can lead to volumetric locking and numerical instability. Therefore, in practical FE analysis, a Poisson’s ratio very close to 0.5 (typically ν = 0.49–0.499) is used to approximate the incompressible response while maintaining numerical stability. This near-incompressible treatment does not alter the underlying constitutive assumption and yields stress–strain responses that are practically indistinguishable from the true incompressible case for the deformation range considered. For this particular case, the Neo-Hookean model reduces to

(14) $$W=\frac{\mu }{2}\left(I_{1}-3\right)=\frac{\mu }{2}\left(\lambda_{1}^{2}+\lambda_{2}^{2}+\lambda_{3}^{2}-3\right)$$

For an incompressible hyperelastic material, the principal Cauchy stresses cannot be obtained by simply differentiating W with respect to *λ*_i_. The correct relation requires a Lagrange multiplier p to enforce the incompressibility constraint:

(15) $$\sigma_{i}=\lambda_{i}\frac{\partial W}{\partial \lambda_{i}}-P,\;i=1,2,3$$

where σ*_i_* is the principal Cauchy stress in the i-th direction, *λ_i_* is the principal stretch ratio in the i-th direction, and P is hydrostatic pressure [Pa] [32,33]. Applying Equation (15) to the incompressible Neo-Hookean model,

(16) $$\sigma_{i}=\mu \lambda_{i}^{2}-P$$

**Figure 1:**
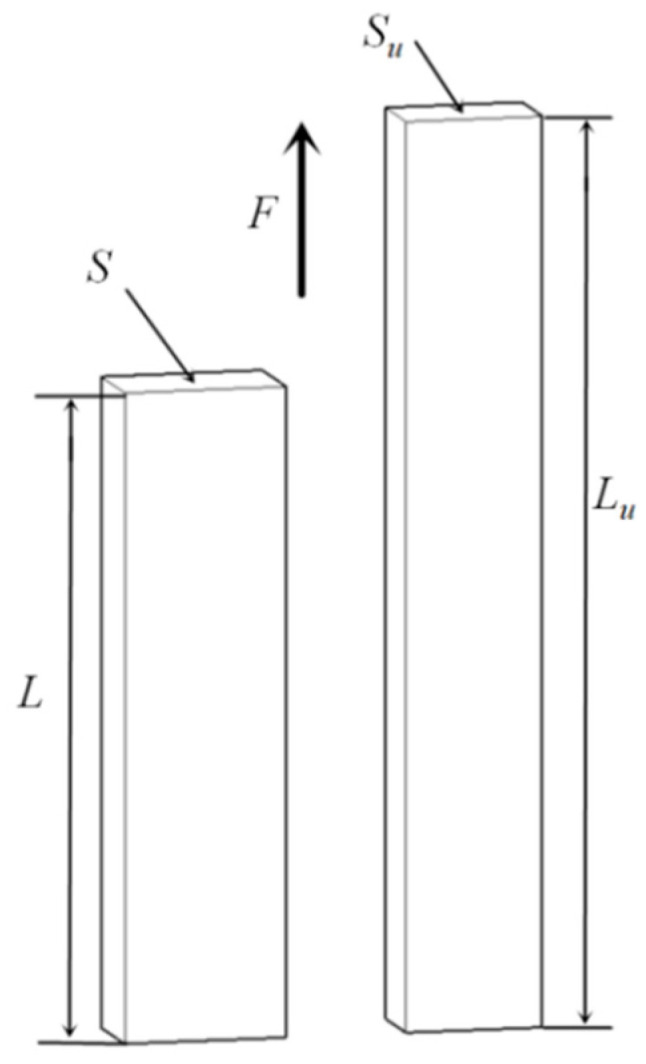
Schematic view of silicone rubber undergoing uniaxial tensile deformation.

The uniaxial tensile deformation of silicone rubber is illustrated in Figure 1, where *L* is the initial length of the rectangular silicone rubber specimen, *S* is the initial cross-sectional area, *F* denotes the applied axial force, *L_u_* is the length after tensile deformation, and *S_u_* is the deformed cross-sectional area.

To characterize the silicone rubber under uniaxial tension, the Cauchy stress *σ*(*UT*) and the stretch ratio *λ* are given by

(17) $$\sigma \left(UT\right)=\frac{F}{S_{u}},\;\lambda =\frac{L_{u}}{L}$$

Owing to the hyperelastic characteristics of silicone rubber, the Cauchy stress does not vary linearly with the stretch ratio. Experimental data on the stress-versus-stretch ratio under uniaxial tension were obtained. By fitting the experimental data to the Cauchy stress expression of the Neo-Hookean model, the values of the material parameters in the Neo-Hookean constitutive model for silicone rubber can be ascertained.

## 3. Uniaxial Tensile Test

The tensile deformation of silicone rubber is influenced by environmental temperature, humidity, and tensile testing parameters. Uniaxial tensile tests on silicone rubber follow national testing standards [7]. The mechanical characterization covered three types of tests: uniaxial tension, equibiaxial tension, and planar tension. The uniaxial test is the basic building block for constructing hyperelastic constitutive models [34]. It should be noted that the equibiaxial and planar tension stress–stretch responses presented in this study are not obtained from independent biaxial or planar experiments. Rather, they are derived from the same uniaxial tensile test data under the assumptions of material incompressibility and isotropy. Over the small-to-moderate strain regime, the Neo-Hookean model yields predictions consistent with Treloar’s classic natural rubber data: the equibiaxial stress is approximately twice the uniaxial stress, and the planar tension response falls between these two curves [35]. At the same strain level, the three stress components relate to each other as

(18) $$\delta_{PT}=1.3\delta_{UT},\;\delta_{BT}=2\delta_{UT}$$

where *δ_UT_* is the uniaxial tensile stress, *δ_PT_* the planar tensile stress, and *δ_BT_* the equibiaxial tensile stress.

During each test, one end of the specimen stays fixed while the other gets pulled at a constant speed. A force sensor and a displacement sensor record the stress–strain curve. These specimens have a dumbbell shape, where the central portions, used for tensile and fracture tests, represent the effective gauge length.

**Figure 2:**
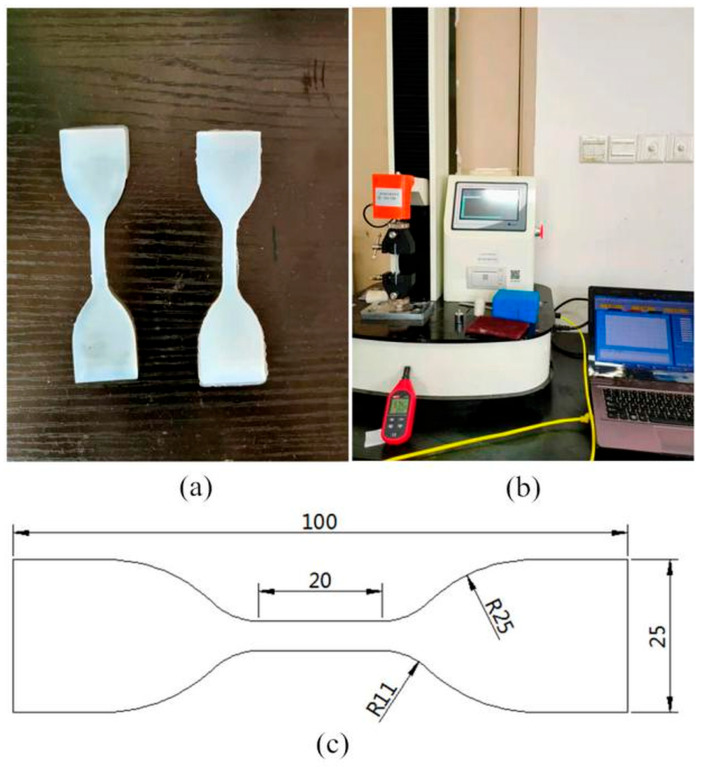
(**a**) Silicone rubber specimens for uniaxial tensile testing. (**b**) Uniaxial tensile test setup. (**c**) Dumbbell specimen dimensions (unit: mm).

Figure 2a,c shows how the silicone rubber specimens were prepared. The liquid components (Parts A and B) of Ecoflex 00–30 silicone were weighed using an electronic scale. Parts A and B were then poured into a plastic cup at a 1:1 ratio and stirred thoroughly. The mixture was subsequently placed in a vacuum chamber to remove entrapped air bubbles. A release agent was sprayed onto the assembled mold surface. The degassed liquid silicone was slowly poured into the mold, which was then placed in an oven and cured at 60 °C for 40 min. The molds were 3D printed to conform to GB/T 528-2009 rubber tensile testing standard [36]. The test pieces were made by curing silicone rubber in these molds. The length of the effective gauge section is 20 mm, and the thickness is less than 3 mm.

For the tensile tests we used a ZQ-990LB series machine (Zhiqu Precision Instrument Co., Ltd., Dongguan, China, Figure 2b), with a load range of 0–50 N and a resolution of 0.001 N—well within the standard’s requirements. The ambient temperature was held at 23 ± 5 °C and relative humidity between 30% and 60%. The actual recorded experimental conditions were 25 ± 3 °C and 57 ± 5% relative humidity.

The silicone rubber specimens were divided into two groups: one group underwent uniaxial tensile tests, and the other group was subjected to uniaxial tensile fracture tests. The tensile speed for the fracture tests was set at 500 mm/min, and the tests were continued until specimen fracture occurred within the initial gauge length. With an initial gauge length of 20 mm, this corresponds to a nominal strain rate of approximately 0.42 s^−1^ (25 min^−1^). The stretch ratios for the uniaxial tensile tests were set at 1–1.5, 1–2, 1–2.5, and 1–3. Under identical experimental conditions, the tensile force versus stretch ratio data for the Ecoflex 00-30 silicone rubber specimens were recorded, and the average value of five test repetitions was taken for each experimental group.

**Figure 3:**
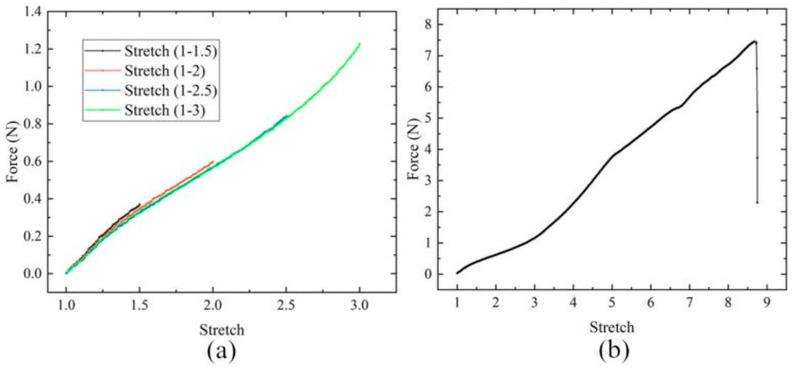
(**a**) Comparison of measured force–stretch curves for silicone rubber at four different stretch ranges. (**b**) Force–stretch curve recorded during the uniaxial tensile test to failure.

Figure 3a presents the uniaxial tensile test curves for silicone rubber specimens at λ = 1–1.5, 1–2, 1–2.5, and 1–3 (corresponding to tensile strain rates of 50%, 100%, 150%, and 200%, respectively). The relationship between the stretch ratio and strain for silicone rubber can be established; in the following analysis, the strain variable is represented by the stretch ratio. The silicone rubber deforms roughly linearly within stretch ratio ranges of 1–1.5 and 1–2. Even at a tensile strain of 100%, the force–stretch ratio curve stays more or less linear. Once the stretch ratio reaches 1–2.5 and 1–3, the material enters a regime of noticeable elastic strain, and the tensile curves turn nonlinear. The tensile test curves for Ecoflex 00-30 agree well across different stretch ratios. This tells us that the material’s elasticity stays stable and uniform over the range we tested.

Figure 3b shows the failure response of the silicone rubber specimens under uniaxial tension. It can be observed that when the stretch ratio is low, the tensile force increases gradually. As the stretch ratio increases, the rate of force increase also rises, indicating that the failure test curve of the silicone rubber exhibits a high degree of nonlinearity. The Ecoflex 00-30 silicone rubber, as a hyperelastic material, reaches failure at a stretch ratio of λ = 8.68.

**Figure 4:**
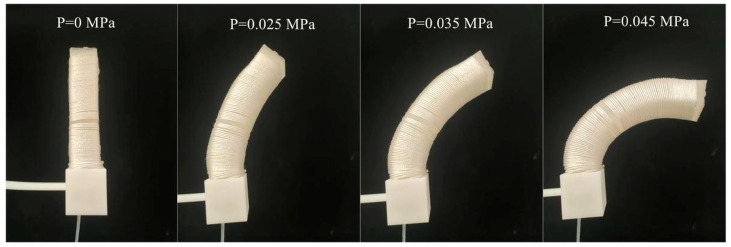
Bending deformation process of soft actuator.

To demonstrate the practical application of the fitted constitutive parameters, a fluid–structure interaction (FSI) simulation of the fiber-reinforced soft actuator was performed. As shown in Figure 4, the simulation captures the bending deformation of the actuator under increasing input air pressure. The bending angle of the fiber-reinforced soft actuator increases gradually with the input air pressure P. As the input air pressure rises to 0.045 MPa, the actuator generates a bending angle of 90°. It should be noted that this simulation serves as a numerical demonstration of how the identified material parameters can be integrated into an actuator-level model, rather than as an experimental validation of the constitutive model itself. Experimental validation of the actuator bending response against physical prototypes is an important direction for future work.

## 4. Fitting of Tensile Test Data in Multiphysics

### 4.1. Fitting of Uniaxial Tensile Test Data

From the force–stretch ratio curves obtained in the uniaxial tensile tests described above, it is clear that silicone rubber exhibits significant tensile deformability and a clearly nonlinear force–stretch ratio response. To design and mechanically analyze soft actuators, it is important to understand the tensile and deformation properties of the silicone rubber under various loading conditions. The stress–strain behavior of the silicone rubber was then fitted and optimized to the Neo-Hookean model using the optimization module in COMSOL Multiphysics (version 5.6) to obtain the parameters of the constitutive model of the Ecoflex 00-30 silicone rubber. With these parameters, we then had a good basis in materials to build from, which can be used in structural optimization, mechanical modeling and accurate pneumatic soft actuator control.

The parameter identification was formulated as a nonlinear weighted least-squares optimization problem. It is important to distinguish between the terms “fitting” and “identification” used in this study: fitting refers to the numerical minimization of the objective function to obtain optimal parameter values, while identification encompasses the broader inverse problem framework, including model selection, identifiability analysis, and reliability assessment. The objective function minimizes the discrepancy between the experimental stress–stretch data and the Neo-Hookean model prediction:

(19) $$f(\mu )=W_{UT}\sum_{i}{\left[\sigma_{\exp}(\lambda_{i})-\sigma_{\mathrm{mod}el}(\lambda_{i};u)\right]}^{2}+W_{BT}\sum_{i}{\left[\sigma_{\exp}(\lambda_{j})-\sigma_{\mathrm{mod}el}(\lambda_{j};u)\right]}^{2}$$

where W_UT_ and W_BT_ are the weight factors assigned to the uniaxial and equibiaxial tension datasets, respectively, and σ_model_(λ; μ) follows from the Neo-Hookean stress expression derived in Section 2.3. The identification problem satisfies Hadamard’s conditions. Existence follows from the continuity of *f*(*μ*) on a compact interval. Uniqueness is guaranteed because the Neo-Hookean stress depends linearly on *μ*, making the single-regime objective quadratic (convex) and therefore globally convex for the piecewise formulation. Stability is ensured by the Levenberg–Marquardt damping parameter, which regularizes the Hessian approximation when the Jacobian is near-singular.

**Figure 5:**
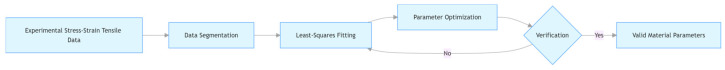
Schematic workflow diagram for fitting tensile test data.

As shown in Figure 5, the Multiphysics Optimization Module enables parameter estimation for constitutive models by solving a nonlinear least-squares problem. The first step is to create a 0D model and add an optimization interface, defining the stretch ratio λ as the x-axis parameter. The 0D model represents a constitutive model fitting analysis that is specific to the silicone rubber material and independent of any particular geometry. Next, the stress–stretch ratio relationship described by the Neo-Hookean model is defined in the optimization module according to Equation (16). The processed stress–stretch ratio data from the uniaxial tensile tests of silicone rubber are then input into the optimization module. The minimization was performed using the Levenberg–Marquardt algorithm, a gradient-based solver optimized for least-squares problems that combines the robust convergence of gradient descent with the rapid local convergence of the Gauss–Newton method. During the optimization process, a global least-squares objective function is added, with the stretch ratio λ specified as the measured parameter and the uniaxial tensile stress–stretch expression Equation (16) designated as the fitting function for the experimental data. The minimization was performed using the Levenberg–Marquardt algorithm, a gradient-based method that achieves quadratic convergence near the minimum by interpolating between gradient descent and Gauss–Newton steps. The objective function is C^2^-smooth with respect to μ, ensuring well-defined gradients and approximate Hessians. The initial guess for the shear modulus was set to *μ*_0_ = 3000 Pa, with lower and upper bounds of 100 Pa and 20,000 Pa, respectively. The number of experimental data points in each fit ranged from approximately N = 100 to N = 500 depending on the stretch range. The optimization converged when the relative change in the objective function fell below 1 × 10^−6^, which typically required 15–30 iterations per regime. To verify global optimality, the optimization was repeated from five random initial guesses (*μ*_0_ = 500–10,000 Pa); all runs converged to the same μ, confirming the absence of local minima. The total computational cost was less than 2 s on a standard desktop computer. This inverse analysis framework follows standard parameter identification methodology and is independent of any specific software implementation. The procedure can be reproduced in any numerical computing environment (MATLAB, Python/SciPy, Abaqus, COMSOL, etc.).

In the optimization interface, the force–stretch ratio data from the uniaxial test were fitted using the Neo-Hookean constitutive model and the first Cauchy stress expression derived. To perform the fitting analysis in the optimization module, the stress–stretch ratio data from the uniaxial tensile test must first be computed using Equation (11), and the corresponding stress–stretch ratio curve must be plotted [37].

Based on *σ*(F), the Neo-Hookean stress–stretch relationship was established in the optimization module. The reduced stress–stretch data from the uniaxial test were input into the optimization module. The stretch ratio λ and a global least-squares objective function were defined, and the stress–stretch ratio data were fitted using the Neo-Hookean model to obtain the material parameters of the silicone rubber constitutive model.

### 4.2. Uniaxial Tension and Equibiaxial Tension Fitting Analysis

In practical engineering applications, isotropic silicone rubber materials are subjected not only to axial tensile loads but also to tensile loads in other directions. Therefore, uniaxial tensile testing alone cannot fully characterize the mechanical properties of silicone rubber. In the case of silicone rubber used in pneumatic soft actuators, axial tensile deformation is the primary mode, accompanied by minor radial tensile deformation. To obtain more accurate constitutive model parameters, both uniaxial and equibiaxial tension fitting analyses were conducted based on the planar tensile stress expression δ_PT_. In the optimization interface, separate datasets were created for uniaxial and equibiaxial tension. An additional global least-squares objective function was defined, along with the corresponding parameters and numerical sequences. Weight factors were assigned to the column contribution weights of each dataset. By adjusting the weight factors for uniaxial and equibiaxial tension, the fitting accuracy can be modulated and different material parameters can be obtained. Since the fitting parameter μ is obtained from the least-squares fitting within a finite strain range, it corresponds to the secant shear modulus. In the fitting of different tensile test datasets for silicone rubber, the weight factors assigned to each dataset significantly influence the fitting performance.

### 4.3. Piecewise Fitting Analysis

**Figure 6:**
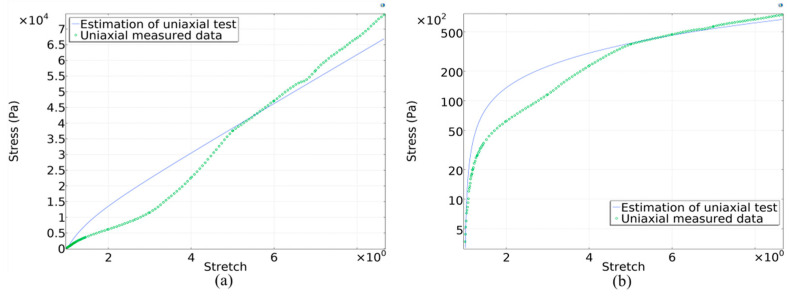
Full-range Neo-Hookean fitting of the uniaxial tensile stress–stretch data: (**a**) fitting of the stress–stretch ratio curve; (**b**) fitting on logarithmic stress coordinates.

As a hyperelastic material, silicone rubber can undergo elastic deformation of 7 to 9 times its original length when subjected to stress. There is a wide range of stretch ratios in tensile tests, and the stress–stretch ratio curve is not a straightforward single nonlinear function. Due to the interactions between molecules and the mobility of the long molecular chains, silicone rubber has several different mechanics over different ranges of stretch ratios. The fitted curve of the Neo-Hookean model is plotted along the stress–stretch ratio curve for uniaxial tensile stress over the range of a stretch ratio of 1–8.6 with the fitted value of the material parameter μ = 7739.9 Pa in Figure 6. In this range, the Neo-Hookean model only agrees with the experimental data at a few points. It performs poorly throughout the entire range of stretch—with the majority of the segments having an obvious deviation. So, when the stretch ratio gets large, the Neo-Hookean model cannot capture the mechanical behavior of silicone rubber with any real accuracy.

For pneumatic soft actuators, silicone rubber undergoes large deformations during actual operation. The Neo-Hookean model, limited in its range of applicability, is insufficient for the design, mechanical analysis, modeling, and precise control of such actuators. Therefore, a piecewise fitting approach was adopted: the uniaxial tensile experimental dataset of silicone rubber was divided into three segments, with each segment fitted independently using the Neo-Hookean model. The first segment corresponds to a stretch ratio range of 1 to 3, the second segment spans 3 to 6, and the third segment ranges from 6 to 8.6.

**Figure 7:**
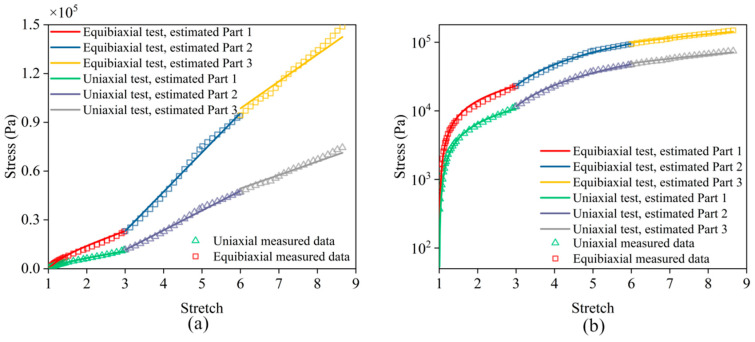
Piecewise parameter identification of the Neo-Hookean model from the uniaxial and equibiaxial tensile stress–stretch ratio curves across the full stretch ratio range under different weight factor conditions: (**a**) three-segment fitting of the stress–stretch ratio curve; (**b**) three-segment fitting on logarithmic stress coordinates.

The uniaxial and equibiaxial tensile datasets were fitted using a three-segment piecewise approach. Different weight factors were assigned to the uniaxial and equibiaxial tensile stress–stretch ratio curves across the various stretch ratio segments. As illustrated in Figure 7, the uniaxial and equibiaxial tensile datasets were divided into three segments: the first segment covers stretch ratios from 1 to 3, the second from 3 to 6, and the third from 6 to 8.6. Within each segment, the weight factors for uniaxial and equibiaxial tension were adjusted based on the fitting accuracy relative to the experimental data. In the first and second segments, the weight factor for uniaxial tension was set lower and that for equibiaxial tension higher; in the third segment, the weight factor for uniaxial tension was set higher and that for equibiaxial tension lower. The three-segment piecewise fitting approach can be rationalized by considering the dominant deformation mechanisms of silicone rubber at different stretch levels. In Regime I (λ = 1–3), the mechanical response is governed primarily by entropic elasticity, where the polymer chains undergo conformational rearrangements with relatively low resistance, resulting in an approximately linear stress–stretch behavior. In Regime II (λ = 3–6), the material enters a transition stage in which the polymer chains begin to align along the loading direction, and the entropic contribution gradually saturates, leading to a moderate stiffening of the response. In Regime III (λ = 6–8.6), the deformation is dominated by molecular orientation and direct stretching of the polymer backbone, causing significant strain stiffening that the Neo-Hookean model, in its standard single-regime form, cannot adequately capture. The piecewise fitting approach therefore reflects these physically distinct regimes by allowing the effective shear modulus to increase progressively with stretch, which is consistent with the underlying molecular mechanisms of rubber elasticity [15,17,35].

To assess the sensitivity of the fitted parameters to the choice of regime boundaries, alternative segmentation schemes were tested. In addition to the nominal boundaries at λ = 3 and λ = 6, two alternative schemes were evaluated: Scheme A (λ = 2.5 and 5.5) and Scheme B (λ = 3.5 and 6.5). The resulting fitted shear moduli for each scheme are summarized in Table 2. It was found that shifting the boundary locations within a reasonable range (±0.5) produced only minor changes in the fitted μ values (typically less than 5% variation), and the fitting accuracy, as measured by the NRMSE, remained comparable across all three schemes. This indicates that the fitting accuracy is primarily governed by the assignment of weight factors to the uniaxial and equibiaxial tension datasets within each regime, rather than by the precise locations of the regime boundaries.

It is important to clarify the scope and limitations of the piecewise fitting approach presented in this study. The piecewise Neo-Hookean model with regime-dependent shear moduli is intended as an empirical engineering tool for interpolation within the calibrated stretch range (λ = 1–8.6). Its primary advantage lies in providing a simple, implementation-ready description of the monotonic hyperelastic response of Ecoflex 00-30 silicone rubber across the full deformation range, using a constitutive model that is natively available in most finite element codes without requiring user-defined subroutines.

However, several limitations should be acknowledged. First, the piecewise parameters are not intended for extrapolation beyond the calibrated stretch range, as the Neo-Hookean model does not capture the limiting chain extensibility that governs behavior near rupture. Second, the multiaxial predictive capability of the piecewise approach has not been independently validated against dedicated equibiaxial or planar tension experiments, since the equibiaxial responses used in the optimization were derived from the same uniaxial data under assumptions of incompressibility and isotropy. Third, the piecewise formulation introduces discontinuities in the tangent modulus at the regime boundaries, which may affect convergence in dynamic FE simulations. Fourth, the approach is limited to monotonic loading and does not capture rate-dependent effects, viscoelasticity, hysteresis, or the Mullins effect.

With piecewise fitting and adjusted weight factors across different stretch segments, the Neo-Hookean model matched the data far better. The fitted curves more accurately model the mechanical response of silicone rubber—this provides a theoretical framework to design pneumatic soft actuators, to analyze their mechanics, to construct kinematic models of the actuators, and to precisely control the actuators. For applications requiring a unified, physically consistent constitutive law with predictive capability across arbitrary loading paths and deformation modes, more advanced models such as the Gent model or the Arruda–Boyce eight-chain model would be more appropriate. The exploration of such unified models for Ecoflex 00-30 is identified as an important direction for future work.

### 4.4. Modeling of Soft Actuators

A soft actuator bends because the applied air pressure and the silicone rubber’s mechanical response work together—it is a coupling problem. In practice, when you design and test these actuators, measuring the stress and deformation of the silicone rubber directly during operation is hard. So is analyzing the stress and strain distribution inside the actuator. To get around this, we built soft actuator models with different structural parameters in Multiphysics and ran fluid–structure interaction (FSI) simulations. The results let us adjust and optimize the structural parameters to improve how the actuator moves. The actuator geometry consists of a rectangular silicone rubber body with an internal air chamber. The overall actuator length is 100 mm, with a width of 20 mm and a height of 15 mm. The internal chamber has a semicircular cross-section with a radius of 5 mm, extending along the entire length of the actuator body. The wall thickness is 2 mm. A reinforcement layer (fiber winding) is modeled as a thin elastic shell surrounding the actuator body, with a fiber winding angle of approximately 45° relative to the longitudinal axis.

**Figure 8:**
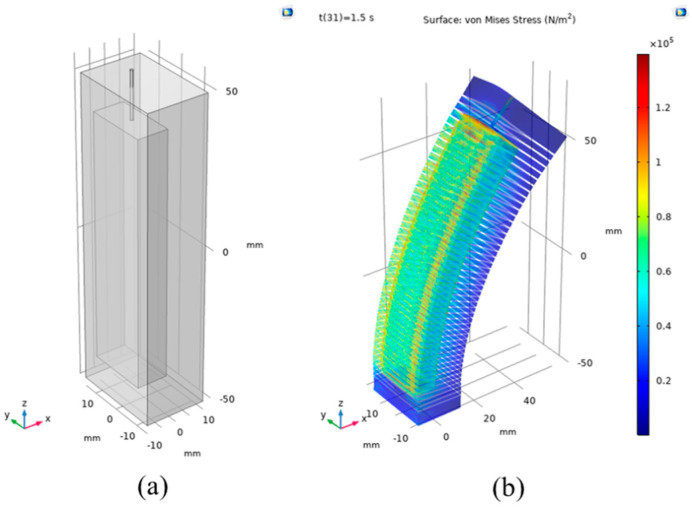
(**a**) Soft actuator model. (**b**) Soft actuator bending deformation.

As shown in Figure 8, a three-dimensional model of the soft actuator was created in COMSOL, incorporating the Solid Mechanics module and the Fluid Mechanics module. The boundary conditions were defined as follows: the base of the actuator was fixed (zero displacement) to represent the clamped end, while all other surfaces were free. A uniform internal pressure was applied to the inner surfaces of the air chamber, ramped from 0 to 0.045 MPa. The silicone rubber was modeled using the incompressible Neo-Hookean formulation derived in Section 2.3. For the numerical FE implementation, a Poisson’s ratio of ν = 0.495 was used to approximate incompressible behavior while avoiding volumetric locking—a standard practice in FE analysis of nearly incompressible materials. The material parameters (shear modulus μ) were taken from the piecewise fitting results in Section 4.3. The fiber reinforcement was modeled as a linear elastic material with Young’s modulus E = 1 GPa. The model was discretized using tetrahedral elements with a maximum element size of 3 mm in the silicone rubber body and 1.5 mm in the chamber wall region, resulting in approximately 50,000 degrees of freedom. A mesh convergence study was performed to ensure that the bending angle results were insensitive to further mesh refinement. The model matches the actual fabricated dimensions.

The actuator material is silicone rubber. The fiber-reinforced actuator is a composite structure in which the matrix material (silicone rubber) maintains isotropy, the fiber is anisotropic, and the reinforcement comes from embedded fibers that provide directional constraints [38]. Using the fitted constitutive parameters for Ecoflex 00-30 from the stress–strain analysis, we assigned the Neo-Hookean model to the material properties to set up the soft actuator. By bringing multiple physical fields into the simulation and defining a convergence criterion, we could simulate how the fiber-reinforced soft actuator deforms under air pressure.

For the numerical demonstration, the simulated bending angle at *p* = 0.045 MPa was compared with the experimental bending angle observed from the fabricated actuator under the same pressure (Figure 4). The simulation predicted a bending angle of approximately 90°, which is in reasonable agreement with the experimental measurement. However, we acknowledge that this represents a single-point comparison and that systematic validation across multiple pressure levels and actuator geometries is necessary to fully assess the predictive capability of the piecewise material parameters. Such a comprehensive validation study is identified as important future work.

## 5. Conclusions

The elementary characteristics of silicone rubber as a pneumatic soft actuator material, the hyperelastic Neo-Hookean model, uniaxial tensile test, and material parameter fitting have been discussed. The computational cost of the piecewise parameter identification is negligible (less than 2 s total on a standard desktop computer), making the approach practical for routine engineering use.

We selected the phenomenological Neo-Hookean constitutive model—this model is based on a strain energy function—to describe the mechanical behavior. We used a uniaxial tensile test to start constructing the model as that is how the material is used. Testing related to tensile fracture, viscoelasticity, hysteresis, and the Mullins effect can be investigated in future work.

The piecewise Neo-Hookean fitting approach presented here is intended as a practical engineering tool rather than a fundamental theoretical advance. Its primary value lies in providing a simple, implementation-ready description of the hyperelastic response of Ecoflex 00-30 across a wide deformation range (λ = 1–8.6), using a constitutive model that is natively available in most finite element codes. The approach enables soft actuator designers to achieve improved fitting accuracy without adopting more complex constitutive models or developing custom material subroutines. Limitations of the piecewise approach, including the lack of tangent continuity at regime boundaries, the reliance on derived rather than independently measured multiaxial data, and the restriction to monotonic loading, are acknowledged and should be addressed in future work through more unified constitutive models such as the Gent or Arruda–Boyce formulations.

We modeled and fitted the Neo-Hookean model in Multiphysics to capture the behavior of silicone rubber under various loading conditions over a broader stretch range. This resulted in constitutive model parameters of Ecoflex 00-30. These parameters give theoretical basis for the design of pneumatic soft actuators, the investigation of their mechanics, creation of kinematic models and precise control of these actuators.

The key results of the Neo-Hookean fitting and tensile testing are given below.

(1) Using the optimization module, we were able to fit the Neo-Hookean model directly to the tensile test data for silicone rubber and optimize it to determine a set of parameters for this hyperelastic material. For the three-segment piecewise fitting, the μ values are 3725.2 Pa (λ = 1–3), 6198.9 Pa (λ = 3–6), and 8250.0 Pa (λ = 6–8.6), representing improvements of 37.51%, 23.39%, and 10.15% in fitting accuracy over the single-parameter approach. In future research, studying the tensile parameters of different elastic body models such as the Gent model is a promising extension.
(2) For these two tensile datasets (uniaxial and equibiaxial), we created independent parameter sets and numerical sequences for the optimization module and gave weight factors for each dataset. Varying these weight factors resulted in better fit of the model to the experimental data, as well as in different material parameters. The weight of each tensile test dataset is important when fitting multiple sets of silicone rubber data.
(3) Silicone rubber exhibits highly different mechanical properties within ranges of stretch ratios. Standard constitutive models typically are only valid over a limited range and are difficult to apply to large engineering deformations. To this end, we adopted a piecewise fitting method where we divided the tensile test data into several segments and adjusted the weight factors for the uniaxial and equibiaxial directions in each segment. This approach significantly enhanced the accuracy of the fitting of the Neo-Hookean model, and the results represented the actual behavior of the material more accurately. The same approach of piecewise fitting with adjustable weight factors can be applied to other hyperelastic materials, giving a theoretical basis for designing and using materials that undergo large deformations.

## Figures and Tables

**Table 1:** The material parameters.

Ecoflex Series Model	Mixing Time	Curing Time	Elongation at Break	Mixed Viscosity	Shrinkage
00–10	40 min	4 h	800%	14,000 cps	<0.001%
00–20	30 min	4 h	845%	3000 cps	<0.001%
00–30	45 min	4 h	900%	3000 cps	<0.001%
00–40	18 min	4 h	980%	8000 cps	<0.001%

**Table 2:** Sensitivity of fitted shear modulus μ to regime boundary selection.

Boundary Scheme	Regime I μ (Pa)	Regime II μ (Pa)	Regime III μ (Pa)
λ = 2.5/5.5	3680.5	6150.2	8310.8
λ = 3.0/6.0	3725.2	6198.9	8250.0
λ = 3.5/6.5	3755.8	6240.1	8195.3

## Data Availability

The original contributions presented in this study are included in the article. Further inquiries can be directed to the corresponding author.
